# Non-vitamin K antagonist oral anticoagulation usage according to age among patients with atrial fibrillation: Temporal trends 2011–2015 in Denmark

**DOI:** 10.1038/srep31477

**Published:** 2016-08-11

**Authors:** Laila Staerk, Emil Loldrup Fosbøl, Kasper Gadsbøll, Caroline Sindet-Pedersen, Jannik Langtved Pallisgaard, Morten Lamberts, Gregory Y. H. Lip, Christian Torp-Pedersen, Gunnar Hilmar Gislason, Jonas Bjerring Olesen

**Affiliations:** 1 Department of Cardiology, Herlev and Gentofte University Hospital, 2900 Hellerup, Denmark; 2 Department of Cardiology, Rigshospitalet, 2100 Copenhagen Ø, Denmark; 3 The Danish Heart Foundation, 1127 Copenhagen K, Copenhagen, Denmark; 4 University of Birmingham Institute of Cardiovascular Sciences, City Hospital, Birmingham B18 7QH, United Kingdom; 5 Institute of Health, Science and Technology, Aalborg University, 9100 Aalborg, Denmark; 6 Faculty of Health and Medical Sciences, University of Copenhagen, 2200 Copenhagen N, Denmark; 7 The National Institute of Public Health, University of Southern Denmark, 1353 Copenhagen K, Denmark

## Abstract

Among atrial fibrillation (AF) patients, Danish nationwide registries (2011–2015) were used to examine temporal trends of initiation patterns of oral anticoagulation (OAC) treatment according to age. Overall, 43,299 AF patients initiating vitamin K antagonists (VKA) (42%), dabigatran (29%), rivaroxaban (13%), or apixaban (16%) were included with mean age (SD) 72.1 (11.3), 71.5 (11.0), 74.3 (11.1), and 75.3 (11.1) years, respectively. Patients aged ≥85 years comprised 15%. Trend tests showed increase in patients ≥85 years initiating OAC (*p* < 0.0001). VKA usage decreased from 92% to 24% (*p* < 0.0001). This decrease was independent of age. Dabigatran was the most common non-VKA OAC (NOAC) (40% users), but usage decreased from 2014 until study end (6%) (*p* < 0.0001). Apixaban was the most used OAC at study end (41%), in particular among those ≥85 years (44%). Compared with patients aged <65 years, the odds ratios associated with initiating VKA, dabigatran, rivaroxaban, or apixaban for patients aged ≥85 years were 0.81 (95% CI 0.75–0.86), 0.65 (95% CI 0.60–0.70), 1.52 (95% CI 1.38–1.67), and 2.09 (95% CI 1.89–2.30), respectively. In conclusion, substantial increase in NOAC usage has occurred. Increasing age was associated with upstart of rivaroxaban or apixaban with reference to age <65 within the specific agent.

Vitamin K antagonists (VKA) have for decades been the only oral anticoagulation (OAC) agent available for stroke prevention in patients with atrial fibrillation (AF), but recently, the landscape for stroke prevention has rapidly changed by the introduction of the non-VKA oral anticoagulants (NOACs)^1^,^2^,^3^. In Denmark, dabigatran was the first NOAC approved for stroke prevention (22 August 2011), followed by rivaroxaban (6 February 2012) and apixaban (10 December 2012). In clinical studies investigating NOACs, elderly patients have been underrepresented, yet it is uncertain into what extent the NOACs are used in elderly^4^,^5^,^6^,^7^. Recent European consensus guidelines from 2015 recommend oral FXa inhibitors (rivaroxaban, apixaban, or edoxaban) over VKAs in the elderly if CrCl >15 ML/min, and in Denmark, we follow the European guidelines, but with the modification that VKA is also recommended if time in therapeutic range ≥70%^8^. The reasons given for the European recommendation of oral FXa inhibitors are the lower incidence of intracranial haemorrhage, the favourable overall efficacy and safety, and the lack of routine monitoring^9^. However, more data are needed to clarify the use of OAC according to age, e.g. how do we best treat octogenarians^3^.

The main objective of this study was to examine temporal trends between 2011 and 2015 of the initiation patterns of NOACs from a nationwide cohort of AF patients in Denmark, and to examine these temporal trends in relation to age.

## Results

### Study population

**Figure 1:**
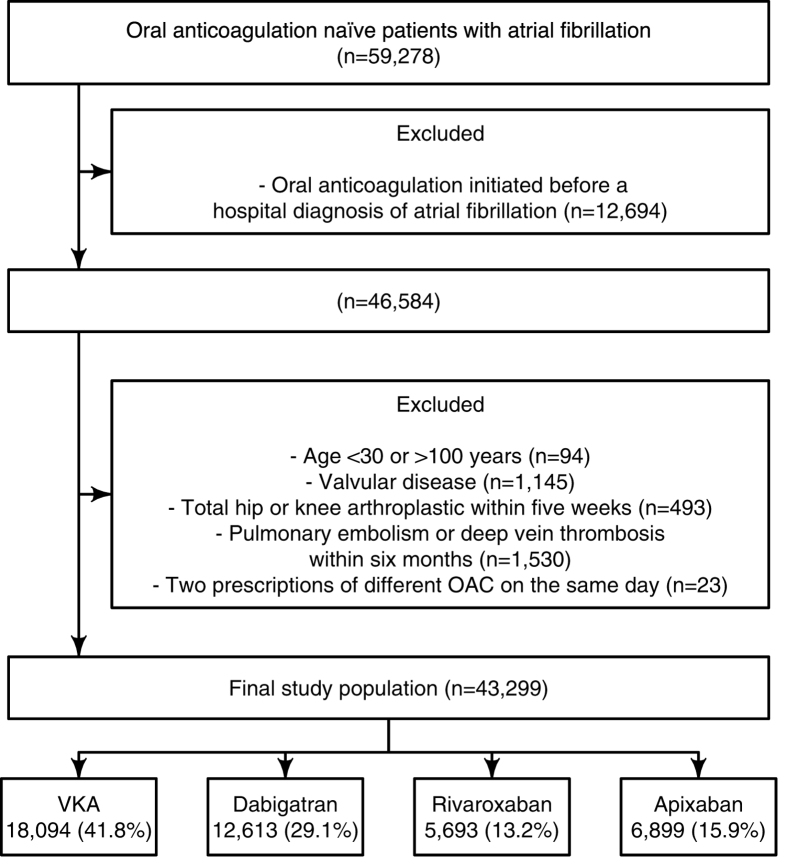
Selection of the study population. Selection of the study population during the study period from 22 August 2011 to 31 December 2015.

Figure 1 shows the selection of the study population. The final study population comprised 43,299 AF patients initiating VKA (41.8%, n = 18,094), dabigatran (29.1%, n = 12,613), rivaroxaban (13.2%, n = 5,693), or apixaban (15.9%, n = 6,899).

Table 1 presents baseline characteristics of the study population according to initiated OAC treatment regimen. Overall, patients initiating dabigatran were youngest (71.5 years, SD 11.0), whereas patients initiating apixaban were oldest (75.3 years, SD 11.1). The distribution of age in the cohort was: 21.0% were <65 years of age, 33.6% were 65 to 74 years, 30.0% were 75 to 84 years, and 15.5% were ≥85 years of age. Low dose of dabigatran, rivaroxaban, and apixaban treatment was initiated in 40.5%, 29.2%, and 36.9%, respectively.

### Temporal trends from 2011 to 2015

**Figure 2:**
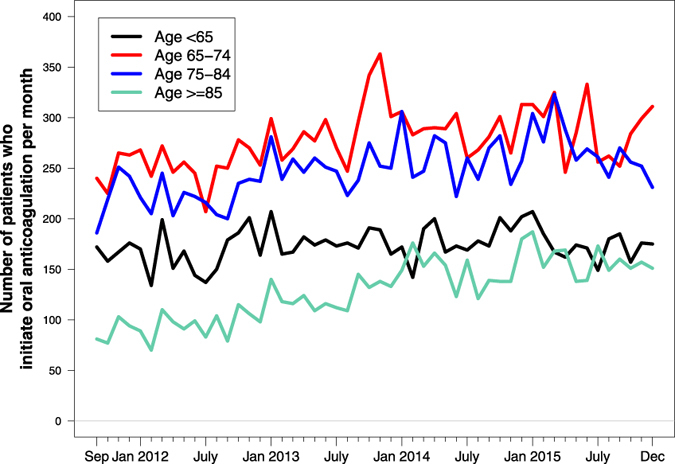
Number of AF patients who initiate oral anticoagulation per month according to age.

Figure 2 shows numbers of patients initiating OAC treatment per month in each age group. The use of OAC increased significantly among AF patients aged <65, 65 to 74, and ≥85 years (*p* < 0.0001, 0.0002, and <0.0001 for increasing trend per year from 2012, respectively), whereas for patients aged 75 to 84 years, number of patients initiating OAC treatment stayed approximately the same per year throughout the study period (*p* = 0.82 for trend per year from 2012).

**Figure 3:**
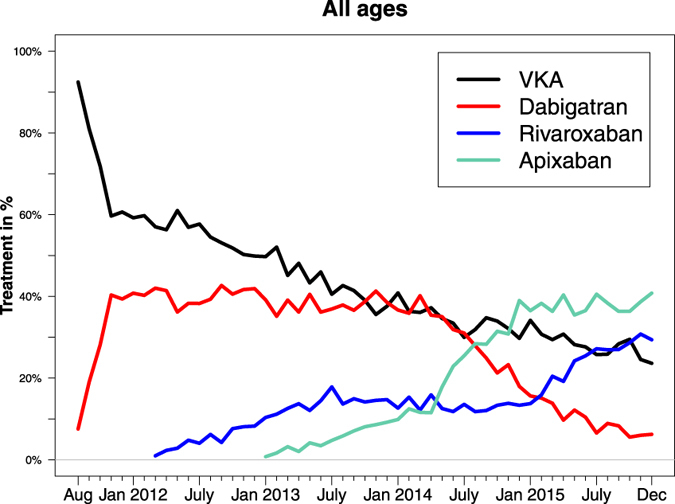
Time trends from 2011 to 2015. Percentages per months for first-time initiators of vitamin K antagonists (VKA), dabigatran, rivaroxaban, and apixaban from 22 August 2011 to 31 December 2015.

Figure 3 illustrates the pattern of initiation of OAC treatment from August 2011 to December 2015. Initiation of VKAs decreased over time—in January 2012, 59% initiated VKA and in December 2015 this was 24% (*p* < 0.0001 for decreasing trend). The utilization of dabigatran increased within a couple of months since its introduction to the market in August 2011. A fairly constant level of dabigatran utilization was seen from December 2011 of approximately 40% until a marked decrease began in 2014 (*p* = 0.04 for trend from December 2011 to December 2013; *p* < 0.0001 for decreasing trend since February 2014). Apixaban was the most used OAC at study end with 41% of patients initiating this OAC treatment (*p* < 0.0001 for increasing trend since January 2013). Since the introduction of rivaroxaban, its usage has steadily increased and at study end 29% of all patients initiating OAC treatment received rivaroxaban.

### Time trends within different age groups

**Figure 4:**
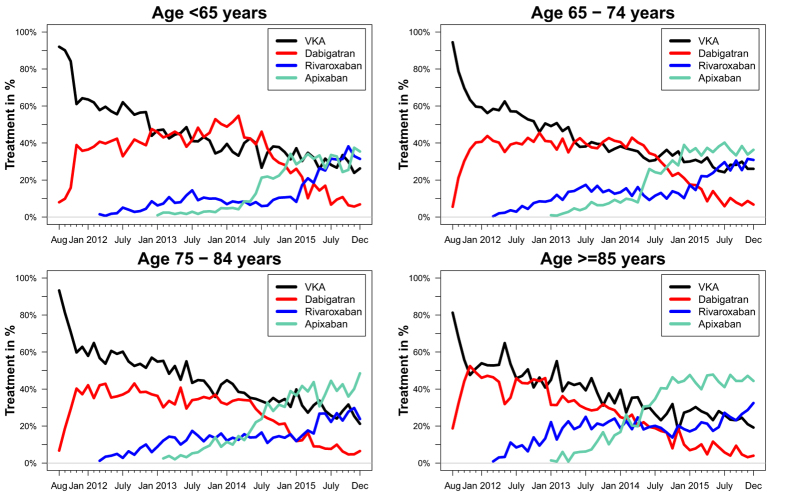
Time trends from 2011 to 2015 according to age. Percentages per months for first-time initiators of vitamin K antagonists (VKA), dabigatran, rivaroxaban, and apixaban according to age groups.

Figure 4 shows the initiation pattern(s) of OAC treatment in relation to the different age groups. Among patients aged <65 years, the utilization of dabigatran increased significantly from August 2011 and peaked in March 2014, where 55% initiated dabigatran (*p* < 0.0001 for increasing trend). From March 2014, the utilization of dabigatran decreased rapidly and in December 2015, only 7% initiated dabigatran (*p* < 0.0001 for decreasing trend), while the most used OAC treatment in December 2015 was apixaban among patients aged <65 years with 35% initiating this drug.

Among patients aged ≥85 years the utilization of VKAs decreased gradually during the study period (*p* < 0.0001 for decreasing trend), and the same was seen for dabigatran (*p* < 0.0001 for decreasing trend from November 2011 to study end). In contrast, an increased use was seen for both rivaroxaban (*p* < 0.0001 for increasing trend from February 2012 to study end) and apixaban (*p* < 0.0001 for increasing trend from January 2013 to study end). Apixaban was the most used drug from July 2014 until study end with approximately 44% initiating apixaban during this period.

### Odds for initiating VKAs, dabigatran, rivaroxaban, or apixaban according to age

Table 2 presents the odds ratios for initiating VKAs, dabigatran, rivaroxaban, or apixaban according to age group using patients aged <65 years as reference group. The odds for initiating VKAs or dabigatran decreased significantly with advancing age. In contrast, the odds for initiating rivaroxaban or apixaban increased significantly with increasing age.

The results from the sensitivity analyses, i.e. when adjusting for various risk factors, substantiated the main results that increasing age was associated with lower odds for initiating VKA or dabigatran and higher odds for initiating rivaroxaban or apixaban.

### Non-included patients

The inclusion criterion was an AF diagnosis followed by the initiation of OAC, but in 12,694 cases, we found that a patient was dispensed a prescription of an OAC agent before being diagnosed with AF at a hospital (VKA, 55.1% (n = 7,001); dabigatran, 27.2% (n = 3,449); rivaroxaban, 10.6% (n = 1346); and apixaban, 7.1% (n = 897)). The median time from an OAC agent was initiated until the AF diagnosis code in The Danish National Patient Registry appeared was 69 days (interquartile range, 24 to 244) for these patients. The 12,694 patients were never included in the final study population shown in Fig. 1.

## Discussion

In this nationwide study including 43,299 OAC-naïve patients with AF, we examined temporal trends in initiation of OAC therapy between 2011 and 2015 according to age. Our principal findings were: (i) the absolute number of patients initiating OAC has increased among patients aged <65, 65 to 74, and ≥85 years; (ii) utilization of VKAs has decreased since the introduction of NOACs; (iii) from 2014 the utilization of dabigatran has decreased, especially among patients aged ≥85 years; and (iv) the utilization of apixaban has increased significantly and was the most used NOAC drug among patients aged ≥85 years since July 2014.

Stroke prophylaxis among high-risk patients, e.g. elderly, is a clinical challenge, and OAC treatment is often underused^10^,^11^,^12^. The suboptimal OAC treatment of elderly patients has been subject to increased focus in recent years as elderly patients are at particular high risk of stroke^9^,^13^,^14^, and prior studies have shown that OAC provides a net clinical benefit among elderly AF patients^14^. In our observational study, we found an increase in the absolute number of patients ≥85 years of age initiating an OAC agent between 2011 and 2015 (see Fig. 2). Our results are encouraging, however it remains to be determined if the proportion of elderly AF patients put on OAC is increasing as well.

This study is methodological similar to one previous published by our group^3^. However, the previous study ended in October 2013 and in this new and updated study, we have a longer study period until 2015 to observe the changes in initiation patterns of OAC treatment. In the previous study, dabigatran was the most used NOAC and overall warfarin was the most used OAC treatment that was initiated among AF patients. However, this pattern has changed since 2013, for example, in December 2015, apixaban was the most used OAC treatment regimen and dabigatran was initiated in less than 10%. We also found that the initiation of VKAs had further declined since our previous study. Reasons for a decline in VKA utilization have been examined by Belen *et al*., and the most common reason for choosing NOAC over VKA in this study was usage problems—for instance, due to interactions with other drugs, diet, etc^15^. Also, a study by AbuDagga *et al*.^16^. reported that older age decreased the probability of initiating dabigatran, but their study ended in 2011 and rivaroxaban and apixaban data were absent. Therefore, we conducted this study to focus on age in a time frame with four OAC treatment regimens being available.

Based on a Danish cohort, we were able to confirm the conclusions of AbuDagga *et al*.^16^. that dabigatran was less used in elderly, but with updated data until 2015, we also show that VKA was less used in elderly. Nevertheless, our results showed that VKA treatment is still fairly used, and in contrast to the NOACs, VKAs have the advantage of being suitable for patients with severe renal impairment (CrCl ≤15 mL/min)^9^.

In The Randomized Evaluation of Long-Term Anticoagulation Therapy (RE-LY) trial, dabigatran 150 mg bid was associated with a notable low relative risk of stroke or systemic embolism compared with warfarin, but the flip side of dabigatran 150 mg bid was an increased risk of gastrointestinal bleedings^5^. We found an initial rise and later decline in dabigatran use over time, in particular among the elderly, who usually need a lower dose of dabigatran (in Denmark and other European countries, dabigatran 110 mg bid is the recommended dose when age ≥ 80 years)^9^. Dabigatran was the first NOAC approved for stroke prevention in AF patients in Denmark and available since August 2011. It is likely that the decline in dabigatran use was influenced by the introduction of rivaroxaban (February 2012) and later apixaban (December 2012) into the market causing an increased focus on novel treatment alternatives. Moreover, the marketing efforts from the pharmaceutical companies could have a role to play. The channelling away from dabigatran has also been observed in another retrospective study from the United States^17^. We also found that the odds were against initiating dabigatran when age increased. Approximately 80% of dabigatran is eliminated by renal clearance, and a condition with impaired renal function is often found among elderly patients^18^. Data from the RE-LY trial showed that renal function was highly correlated with age, and plasma concentration of dabigatran increased with advancing age^19^. In our study patients initiating dabigatran had the lowest frequency of chronic kidney disease (1.9%). We also tested if chronic kidney disease or the parameters in the CHA_2_DS_2_-VASc or the HAS-BLED scores could have confounded the results of low usage of dabigatran in the elderly, but increasing age independently remained a factor associated with non-selection of dabigatran. However, the ESC Thrombosis Working Group and the national Danish recommendation propose that advanced age alone should not exclude the use of dabigatran^9^. In 2016, idarucizumab for dabigatran reversal was approved in Denmark, and this leaves dabigatran with the advantage of being the only NOAC with an available antidote^20^.

In this study rivaroxaban was the only NOAC dosed as only one tablet per day, and in some cases this might have been an attractive option in clinical practice. In the phase III randomised trial of rivaroxaban versus warfarin in AF, rivaroxaban was non-inferior to warfarin for the prevention of stroke/systemic embolism, with similar major bleeding rates^6^.

The Apixaban for Reduction in Stroke and Other Thromboembolic Events in Atrial Fibrillation (ARISTOTLE) trial found that apixaban had a lower relative risk of stroke or systemic embolism, as well as major bleeding and death when compared with warfarin^7^.

A recent published review by Diener *et al*. suggests apixaban as first choice of OAC treatment in AF patients older than 75 years^21^. In concordance with this, we found that advancing age was associated with increased odds of initiating apixaban, and since July 2014, apixaban has been the most used drug among patients ≥85 years of age in our study. The efficacy and safety of apixaban compared with warfarin according to age has been examined in post-hoc analyses on observations from ARISTOTLE trial^4^. The results showed that among patients aged ≥75 years, treatment with apixaban was associated with a lower risk of events (included stroke/thromboembolism, major bleeding, or death from any cause) compared with warfarin with an adjusted hazard ratio of 0.82 (95% confidence interval 0.72–0.93). The authors concluded that among elderly AF patients, the absolute benefit of apixaban outweighed warfarin. Even though no comparable head-to-head randomised trial has examined the efficacy and safety between the NOACs, it is likely that the positive results from the ARISTOTLE trial might have influenced the rapidly increased uptake of apixaban compared with the other NOACs, in particular among elderly AF patients^22^.

In the Apixaban Versus Acetylsalicylic Acid to Prevent Stroke in Atrial Fibrillation Patients Who Have Failed or Are Unsuitable for Vitamin K Antagonist Treatment (AVERROES) trial, apixaban was compared to aspirin in patients where VKA was unsuitable or refused^23^. The results showed that apixaban was superior to aspirin for stroke prevention, with a similar rate of major bleeding.

Within the recent years, it is possible that for whom VKAs have been unsuitable, apixaban would now be initiated instead of aspirin or no OAC therapy, and this could also influence the increase in apixaban utilization that was seen in our study. In particular, this may partly explain the increase in apixaban utilization among the elderly. Also, aspirin is not recommended as stroke prophylaxis in the European AF guidelines since 2010^1^.

In this study we examined temporal trends of NOAC utilization according to age, and future research is warranted to investigate the risk of stroke and bleeding with NOACs compared to VKA among AF patients—in particular among the elderly.

The main strengths of this study essentially consisted of two components: the prospective collection of data and the complete nationwide data. The prospective collection of data enabled us to ascertain how OAC is used in an everyday clinical setting from 2011 to 2015. The Danish registries provided complete nationwide data unaffected by geography, socioeconomic status, and affiliation to the labour market or participation in health insurance programs. Every resident in Denmark is covered by a public tax financed health insurance system, which ensures registration of all hospital admissions and outpatient contacts. In addition, due to partial reimbursement of drug expenses by Danish healthcare authorities, all pharmacies are required to register all claimed drug prescriptions, which ensures complete registration. The Danish registries have been examined for numerous purposes and found accurate for a variety of conditions^24^,^25^,^26^. Relevant to this study; a validation study has shown that a hospital-related AF diagnosis has a positive predictive value of 99%^27^. Another validation study of the Danish National Patient Register by Rix *et al*. from 2012 concluded that the validity of AF is high and may be used for registry-based studies^28^,^29^.

A limitation was the lack of clinical information, such as data on: serum creatinine concentrations, haemoglobin levels, the international normalised ratio levels, body mass index, and other potential confounders that could have influenced our results. Chronic kidney disease and the parameters in the CHA_2_DS_2_-VASc and the HAS-BLED scores were tested as confounders in the logistic regression models, and this did not influence the association between age and initiation pattern of OAC treatment.

We have no information about whether the first OAC prescription was prescribed in primary care (general practitioner) or in secondary care (hospital). Our study population consisted of patients diagnosed with AF at a hospital (both in- and outpatients) before initiating OAC treatment, and the population size was reduced about one fifth (12.600/59.000~20%) due to this approach. A minority of patients might be handled in primary care only, and this imposes a potential selection bias.

Owing to the observational study design, the results from the logistic regression models were limited by the fact that no causative link between age and choice of OAC treatment could be drawn; instead, the results were based on associations. Finally, our results show time trends for the utilization of OAC in Denmark, but the utilization patterns could vary from one country to another.

In conclusion, this study examined the initiation pattern of VKA and NOACs according to age with nationwide data on 43,299 AF patients from 2011 to 2015. We found that OAC has been increasingly used in AF patients aged ≥85 years, the initiation of VKAs has declined gradually since 2011 at the expense of an increase in initiation of NOAC, and with reference to age <65 within the specific agent, the odds ratio of initiating treatment with rivaroxaban or apixaban increased with advancing age.

## Methods

### Data sources

In Denmark all residents are at birth or immigration registered with a unique personal identification number that enables individual-level-linkage between nationwide administrative registers. We cross-linked individual information from three nationwide registries. The Danish National Patient Register keeps data on all hospitalizations including data on outpatients since 1978. The International Classification of Diseases (ICD) (the 8^th^ revision until 1994 and the 10^th^ revision thereafter) is used to code one primary, and if appropriate, any secondary diagnoses for each hospital admission and contact^24^. Data from general practitioners were not available. The Danish National Prescription Register holds information on all prescriptions dispensed from a pharmacy since 1995 with data on Anatomical Therapeutic Chemical (ATC) codes, the day each prescription was dispensed, the package size, and dosages^26^. The Danish Civil Registration System records vital status and date of death^25^.

### Study population

We conducted an observational study and included all hospital-registered AF patients on the day they filled a first-time prescription of VKA, dabigatran, rivaroxaban, or apixaban during the study period from 22 August 2011 to 31 December 2015 (referred to as OAC-naïve AF patients). Patients with a filled OAC prescription prior to study start was not included, i.e. patients with a filled prescription from 1 January 1995 to 21 August 2011. We were able to identify a group of patients who filled a first-time prescription of an OAC drug before being registered with AF at a hospital. These patients were not included in the study population of two reasons: At first, we have no data regarding diagnosis codes from general practitioners, and therefore, it was possible that OAC was prescribed for other reasons than stroke prophylaxis in these patients. Secondly, the AF diagnosis code related to a hospital contact has been validated in a study based on the Danish National Patient Register, and the results revealed a 99% positive predictive value^27^. This high positive predictive value encouraged us to only include patients who had a hospital-related AF diagnosis and subsequently filled an OAC prescription.

The additional exclusion criteria were: (i) age <30 years or >100 years; (ii) valvular AF; (iii) total hip or knee arthroplastics within five weeks before inclusion day; (iv) pulmonary embolism or deep vein thrombosis within six months before inclusion day; or (v) two prescriptions of different OAC claimed at the inclusion day. We incorporated these exclusion criteria to obtain a more homogeneous study population, to ensure OAC was initiated for stroke prophylaxis as chronic treatment, and to avoid misclassification of initiated OAC treatment.

### Concomitant medication and comorbidities

Information on concomitant medicine was obtained from ATC codes of prescriptions dispensed within 180 days prior to the date of inclusion. Comorbidities were identified from ICD codes from hospitalizations up to10 years prior to the inclusion date (see Supplementary Information). The risk of stroke and bleeding was assessed with risk stratification schemes according to CHADS_2_ (congestive heart failure, hypertension, age ≥75 years, diabetes mellitus, stroke/systemic thromboembolism/transient ischaemic attack (2 points)), CHA_2_DS_2_-VASc (CHADS_2_ adding vascular disease, age 65 to 74 years, and female sex, and 2 points for age ≥75 years), and HAS-BLED (hypertension, abnormal renal or liver function, stroke/systemic thromboembolism, previous bleeding, labile international normalised ratio (left out because data are unavailable), elderly (age ≥65 years), and drug (antiplatelet agents, non-steroidal anti-inflammatory drugs)/alcohol abuse) scores^30^,^31^,^32^.

### Definition of age groups

The main covariate of interest was age. We divided the included patients into four age groups according to the age of the patient when initiating OAC—that was age <65 years, 65 to 74 years, 75 to 84 years, and ≥85 years.

### Statistics

Baseline characteristics were reported as numbers with percentages for categorical data and as mean with standard deviation (SD) for continuous data. For comparisons, we performed tests using the Kruskal-Wallis rank sum test on continuous variables (age, CHADS_2_, CHA_2_DS_2_-VASc, and HAS-BLED scores) and Chi-squared test for the categorical variables. OAC prescription patterns per month were plotted as numbers and frequencies of patients initiating treatment with a specific OAC. Temporal trends were estimated with Cochran-Armitage trend tests based on the null hypothesis of no temporal change in utilization patterns within the specific agent. With hypothesis testing we measured how likely no change in data occurred over a period of interest (H0: μ_1_ = μ_2_), and the null hypothesis was rejected if a change over time was concluded. A P value below 0.05 was considered statistical significant, e.g. the null hypothesis was rejected if p < 0.05.

We used sex and calendar year-adjusted logistic regression models to estimate the odds of initiating VKA, dabigatran, rivaroxaban, or apixaban when aged 65 to 74 years, 75 to 84 years, and ≥85 years, respectively, compared with the reference group being patients <65 years of age. Sensitivity analyses were performed to check for possible confounders: (i) adjustments of chronic kidney disease; (ii) adjustments of the parameters in the CHA_2_DS_2_-VASc score; and (iii) adjustments of the parameters in the HAS-BLED score. Finally, we fitted the models with age as a quantitative variable.

Data management and statistical analyses were performed using SAS (version 9.4 for Windows, SAS Institute, North Carolina) and R (version 3.0.2 for Windows, R Foundation for Statistical Computing).

### Ethics

Retrospective registry-based studies do not require approval from the Research Ethics Committee System. The Danish Data Protection Agency had approved use of data for this study (ref. no: 2007-58-0015/GEH-2014-012 I-Suite no: 02720).

## Additional Information

**How to cite this article**: Staerk, L. *et al*. Non-vitamin K antagonist oral anticoagulation usage according to age among patients with atrial fibrillation: Temporal trends 2011–2015 in Denmark. *Sci. Rep.* **6**, 31477; doi: 10.1038/srep31477 (2016).

## Supplementary Material

Supplementary Information

## Figures and Tables

**Table 1:** Baseline characteristics.

	VKA	Dabigatran	Rivaroxaban	Apixaban	*p*-value
N (%)	18094 (41.8)	12613 (29.1)	5693 (13.2)	6899 (15.9)	
Males (%)	10265 (56.7)	6938 (55.0)	2838 (49.9)	3439 (49.8)	<0.001
Age (mean [SD])	72.05 (11.25)	71.54 (11.01)	74.34 (11.14)	75.29 (11.10)	<0.001
Age groups (%)					<0.001
<65	4005 (22.1)	3053 (24.2)	979 (17.2)	1051 (15.2)	
65 to 74	6028 (33.3)	4467 (35.4)	1885 (33.1)	2146 (31.1)	
75 to 84	5671 (31.3)	3507 (27.8)	1656 (29.1)	2156 (31.3)	
≥85	2390 (13.2)	1586 (12.6)	1173 (20.6)	1546 (22.4)	
CHADS_2_ (mean [SD])	1.54 (1.24)	1.40 (1.19)	1.57 (1.25)	1.66 (1.26)	<0.001
CHA_2_DS_2_- VASc (mean [SD])	2.89 (1.64)	2.70 (1.58)	2.99 (1.60)	3.11 (1.60)	<0.001
HAS-BLED (mean [SD])	2.16 (1.22)	2.00 (1.16)	2.14 (1.15)	2.20 (1.19)	<0.001
High dose	—	7503 (59.5)	4028 (70.8)	4352 (63.1)	<0.001
Low dose	—	5110 (40.5)	1665 (29.2)	2547 (36.9)	<0.001
Comorbidities (%)					
Stroke	2633 (14.6)	1950 (15.5)	1024 (18.0)	1429 (20.7)	<0.001
Myocardial infarction	1963 (10.8)	881 (7.0)	353 (6.2)	505 (7.3)	<0.001
Ischemic heart disease	4682 (25.9)	2487 (19.7)	1115 (19.6)	1454 (21.1)	<0.001
Peripheral artery disease	757 (4.2)	310 (2.5)	178 (3.1)	230 (3.3)	<0.001
Heart failure	3522 (19.5)	1818 (14.4)	874 (15.4)	1077 (15.6)	<0.001
Diabetes mellitus	2451 (13.5)	1403 (11.1)	662 (11.6)	885 (12.8)	<0.001
Hypertension	8475 (46.8)	5499 (43.6)	2525 (44.4)	2953 (42.8)	<0.001
Chronic kidney disease	1367 (7.6)	242 (1.9)	208 (3.7)	315 (4.6)	<0.001
Abnormal liver function	303 (1.7)	131 (1.0)	70 (1.2)	102 (1.5)	<0.001
Bleeding	2131 (11.8)	1336 (10.6)	617 (10.8)	912 (13.2)	<0.001
Alcohol abuse	540 (3.0)	396 (3.1)	183 (3.2)	240 (3.5)	0,246
Concomitant medication (%)					
ADP receptor antagonists	1784 (9.9)	1072 (8.5)	580 (10.2)	771 (11.2)	<0.001
Aspirin	7712 (42.6)	4766 (37.8)	2149 (37.7)	2456 (35.6)	<0.001
Dipyridamole	663 (3.7)	333 (2.6)	159 (2.8)	199 (2.9)	<0.001
Non-steroidal antiinflammatory drugs	2668 (14.7)	1882 (14.9)	824 (14.5)	976 (14.1)	0,493
Loop diuretics	3983 (22.0)	1876 (14.9)	1000 (17.6)	1288 (18.7)	<0.001
Beta-blockers	8056 (44.5)	4821 (38.2)	2206 (38.7)	2533 (36.7)	<0.001
Calcium channel blockers	5170 (28.6)	3294 (26.1)	1528 (26.8)	1811 (26.3)	<0.001
Renin-angiotensin system inhibitors	7808 (43.2)	5270 (41.8)	2331 (40.9)	2935 (42.5)	0,011
Digoxin	1327 (7.3)	860 (6.8)	436 (7.7)	426 (6.2)	0,002

Numbers and percentages of first-time initiators of vitamin K antagonists (VKA), dabigatran, rivaroxaban, and apixaban from 22 August 2011 to 31 December 2015.

**Table 2:** The probability of initiating oral anticoagulation according to age.

	Age groups			
	<65	65 to 74	75 to 84	≥85
VKA	Reference	0.93 (0.88–0.98)	1.05 (0.99–1.11)	0.81 (0.75–0.87)
Dabigatran	Reference	0.88 (0.83–0.94)	0.73 (0.69–0.78)	0.65 (0.60–0.70)
Rivaroxaban	Reference	1.20 (1.10–1.31)	1.14 (1.04–1.24)	1.52 (1.38–1.67)
Apixaban	Reference	1.33 (1.22–1.45)	1.49 (1.37–1.63)	2.09 (1.89–2.30)

Odds ratios showing the probability of initiating first-time oral anticoagulation according to age. Odds ratios are estimated with a sex and calendar year-adjusted logistic regression model with the reference group being <65 years of age.
