# Supplementary material for: Non-vitamin K antagonist oral anticoagulation usage according to age among patients with atrial fibrillation: Temporal trends 2011–2015 in Denmark
Source: Sci Rep. 2016 Aug 11;6:31477. doi: 10.1038/srep31477 (PMC4980590; doi:10.1038/srep31477)
Supplement: Supplementary Information [file srep31477-s1.pdf]

Original Research

Non-vitamin K antagonist oral anticoagulation usage according to age among patients with atrial fibrillation: Temporal trends 2011-2015 in Denmark

Laila Staerk*, MD (1), Emil Loldrup Fosbøl, MD, PhD (2,3), Kasper Gadsbøll, MB (1), Caroline Sindet-Pedersen, M.Sc.Pharmacy (1), Jannik Langtved Pallisgaard, MD (1,6), Morten Lamberts, MD, PhD (1), Gregory Y H Lip, MD, professor (4), Christian Torp-Pedersen, MD, DMSc, professor (5), Gunnar Hilmar Gislason, MD, PhD, professor (1,3,5,6), Jonas Bjerring Olesen, MD, PhD (1).

Supplementary Information

Population

Non-valvular atrial fibrillation	*Defined from diagnosis of atrial fibrillation with the absence of diagnosis codes of valvular atrial fibrillation and mitral- or aortic valve surgery.*	Presence of:<br>ICD8: 42793, 42794.<br>ICD10: I48.<br>Absence of:<br>ICD8: 4240, 4241, 39500- 39502, 39508, 39509, 39600-39604, 39608, 39609.<br>ICD10: I05, I06, I080A, I081A, I082A, I083A, Z952, Z954.<br>NCSP: KFKD, KFKH, KFMD, KFMH, KFGE, KFJF.
Vitamin K antagonist	*Defined from ATC-code.*	ATC: B01AA03, B01AA04.
Dabigatran	*Defined from ATC-code.*	ATC: B01AE07.
Rivaroxaban	*Defined from ATC-code.*	ATC: B01AF01.
Apixaban	*Defined from ATC-code.*	ATC: B01AF02.
Hip or knee replacement surgery	*Defined from surgical procedure performed.*	NCSP: KNFB, KNFC, KNGB, KNGC.
Deep venous thrombosis	*Defined from diagnosis.*	ICD10: I801-I803, I808, I809, I821-I823, I828, I829
Pulmonary embolism	*Defined from diagnosis.*	ICD10: I26.

Comorbidities

Stroke/thromboembolism	*Defined from diagnosis of ischemic stroke, transient ischemic attack, or*	ICD10: I63, I64, I74, G458, G459.

	peripheral artery embolism.	
Myocardial infarction	*Defined from diagnosis.*	ICD10: I21, I22.
Ischemic heart disease	*Defined from diagnosis.*	ICD10: I20-I25.
Peripheral artery disease		ICD10: I70
Heart failure	*Defined from diagnosis.*	ICD10: I42, I50, I110, J81.
Diabetes mellitus	*Defined from glucose-lowering medication.*	ATC: A10.
Hypertension	*Defined from combination treatment with at least two classes of antihypertensive drugs: adrenergic α-antagonists, non-loop diuretics, vasodilators, beta-blockers, calcium channel blockers and renin-angiotension system inhibitors.*	ATC: C02A, C02B, C02C, C02L, C03A, C03B, C03D, C03E, C03X, C07B, C07C, C07D, C08G, C02DA, C09BA, C09DA, C02DB, C02DD, C02DG, C07A, C07B, C07C, C07D, C07F, C08, C09BB, C09DB, C09AA, C09BA, C09BB, C09CA, C09DA, C09DB, C09XA02, C09XA52
Chronic kidney disease	*Defined from diagnosis of chronic glomerulonephritis, chronic tubulointestinal nephropathy, diabetic, and hypertensive nephropathy among others.*	ICD10: E102, E112, E132, E142, I120, M300, M313, M319, M321B, N02-N08, N11, N12, N14, N18, N19, N26, N158, N159, N160, N162, N163, N164, N168, Q61, Q613, Q615, Q619.
Abnormal liver function	*Defined from diagnosis of liver chronic liver disease, cirrhosis and hepatitis.*	ICD10: B15-B19, C22, D684C, I982, K70-K77, Q618A, Z944.

Bleeding	*Defined from diagnosis of intracranial bleeding, major gastrointestinal bleeding, respiratory or urinary tract bleeding, and bleeding due to anaemia.*	ICD10: D500, D62, G951A, H052A, H313, H356, H431, H450, I312, I60-I62, I850, I864A, J942, K228F, K298A, K250, K252, K254, K256, K260, K262, K264, K266, K270, K272, K274, K276, K280, K282, K284, K286, K625, K661, K638B, K638C, K838F, K868G , K920, K921, K922, N02, R04, R31, S064, S065, S066, S368D.
Alcohol abuse	*Defined from alcohol-related diagnosis codes or at least one dispensed prescription of an alcohol antagonist drug used to treat chronic alcoholism.*	ICD10: E244, E52, F10, G312, G621, G721, I426, K292, K70, K860, L278A, O354, T51, Z714, Z721. ATC: N07BB.

Concomitant medication

ADP receptor antagonists	*Defined from ATC-code.*	ATC: B01AC04, B01AC22, B01AC24
Acetylsalicylic acid (aspirin)	*Defined from ATC-code.*	ATC: B01AC06, N02BA1.
Dipyridamole	*Defined from ATC-codes.*	ATC: B01AA07.
Non-steroidal anti-inflammatory drugs	*Defined from ATC-codes.*	ATC: M01A, M01AX05.
Loop diuretics	*Defined from ATC-codes.*	ATC: C03C

Beta-blockers	*Defined from ATC-codes.*	ATC: C07A, C07B, C07C, C07D, C07F.
Calcium channel blockers	*Defined from ATC-codes.*	ATC: C08, C09BB, C09DB
Renin-angiotensin system inhibitors	*Defined from ATC-codes.*	ATC: C09AA, C09BA, C09BB, C09CA, C09DA, C09DB, C09XA02, C09XA52
Digoxin	*Defined from ATC-codes.*	ATC: C01A
